# Evaluating the Feasibility of a Dyadic, Touch-Based Multimedia Tablet Intervention and Its Effects on the Caregiver-Patient Relationship Among Individuals With Mild Cognitive Impairment: Qualitative Triangulation Study

**DOI:** 10.2196/75189

**Published:** 2025-08-28

**Authors:** Alexa von Bosse, Anna Thum, Sonja Mahler, Henriette Schulz, Paulina Weuthen, Christophe Kunze

**Affiliations:** 1 Care & Technology Lab Faculty 3: Health, Medical & Life Sciences Furtwangen University Furtwangen Germany; 2 Competence Center Dementia Department Health Ostschweizer Fachhochschule OST St Gallen Switzerland; 3 Faculty 3: Health, Medical & Life Sciences Furtwangen University Furtwangen Germany

**Keywords:** mild cognitive impairment, caregivers, relationship, feasibility, tablet-based intervention, multimedia intervention, dyadic intervention, artificial intelligence

## Abstract

**Background:**

Approximately 20% of the global population is affected by mild cognitive impairment (MCI), with around 15% progressing to dementia within 2 years. Touch-based multimedia applications can support cognitive, social, and physical functioning, potentially enhancing daily life and strengthening caregiver-patient relationships through shared engagement. Although interest in dyadic, technology-assisted interventions is increasing, empirical evidence on their feasibility and acceptability in home-based settings remains scarce. In particular, little is known about their impact on caregiver–care recipient dynamics and the factors that facilitate or hinder their use.

**Objective:**

We aimed to evaluate the feasibility of a dyadic, tablet-based multimedia intervention for individuals with MCI and their caregivers in a home setting, focusing on user experiences, use barriers and facilitators, and the intervention’s impact on the caregiver-patient relationship.

**Methods:**

We applied qualitative triangulation, combining naturalistic observations and semistructured interviews. Data were analyzed using qualitative content analysis. The intervention was codeveloped by a multidisciplinary team and implemented as a user-centered, tablet-based modular platform with customizable cognitive, physical, and interactive exercises.

**Results:**

We recruited a total of 40 participants, comprising 20 (50%) individuals with MCI and their 20 (50%) caregivers. Our study confirmed the feasibility of a touch-based multimedia intervention for both groups. Despite initial challenges with navigation and touch interfaces, most participants demonstrated increased confidence and competence, particularly with tailored caregiver support. Instances of enhanced communication and emotional connection were described by care partners and then became visible during their interaction with the intervention. Shared moments of laughter, mutual encouragement, and coordinated task execution indicated that the intervention could create opportunities for relational closeness. Biography-related tasks proved particularly effective, stimulating meaningful conversation and storytelling that facilitated the sharing of personal memories and experiences. While some dyads experienced occasional tensions due to impatience, differing expectations, or dominant behavior by one partner, the overall atmosphere was one of cooperation, support, and adaptability. The intervention demonstrated how technology can serve as a tool to facilitate shared experiences, promote cognitive engagement, and enrich interpersonal relationships in everyday caregiving contexts.

**Conclusions:**

Our findings show that the home-based implementation of a dyadic, touch-based multimedia intervention can be feasible for individuals with MCI and their caregivers. A key finding is that biographically oriented content fosters caregiver-patient relationships by acting as a catalyst for personal dialogues and collective reminiscence. These interactions enhance emotional intimacy and mutual understanding, highlighting the potential of technology-driven interventions in dementia care. The intervention incorporated multiple domains, including cognitive stimulation, physical activation, and communicative-social interaction, all of which proved highly promising. In addition, the regular implementation of the intervention in home settings appears to be a realistic and achievable approach.

## Introduction

### Epidemiology of Dementia and Mild Cognitive Impairment

Approximately 50 million people are living with dementia, and the prevalence of dementia is projected to increase to 152 million by 2050 [1]. Mild cognitive impairment (MCI) is characterized by a decline in cognitive function that is more pronounced than normal aging but not as severe as dementia, often manifesting as memory impairment [2]. Approximately 15% of individuals with MCI progress to dementia within 2 years [3]. While MCI does not typically lead to significant impairments in activities of daily living [2], individuals with dementia often experience substantial limitations in performing everyday tasks [4].

### Tablet-Based Interventions and Dyadic Interaction

Given the potential progression to dementia and the increasing prevalence of MCI with advancing age, early interventions, such as cognitive training applications, are particularly important in an aging society [3]. Maintaining cognitive, social, and physical functioning is essential for preserving quality of life. Assistive technologies, including touch-based multimedia applications, have been shown to support daily activities, enhance quality of life, and reduce the financial burden of inpatient care [4]. Cognitive stimulation, such as through touch-based multimedia applications, may contribute to the management of MCI and mitigate the cognitive decline of affected individuals [5].

While tablet-based interventions for individuals with MCI may offer cognitive and physical benefits, research suggests that these interventions, particularly those incorporating reminiscence activities and fostering dyadic interactions between caregivers and individuals with MCI in both inpatient and home settings, can also enhance communication and social engagement [6]. Moreover, tablet-based interventions provide an engaging and enjoyable means of interaction for individuals with MCI, potentially strengthening social relationships [7].

Some interventions incorporate activity content such as photos, audio elements, and videos, which have been shown to facilitate memory activation and increase social engagement. However, research on the effects of technology-based interventions on caregiver-patient relationships and social interaction in individuals with cognitive impairments remains limited [6]. Nevertheless, existing evidence indicates a reciprocal relationship between the perceived burden of caregiving and the caregiver-patient relationship. While the ongoing cognitive decline of care recipients can heighten role strain for caregivers, dyadic relational resources, such as mutuality, have been shown to alleviate this burden [8].

To strengthen the dyadic relationship, it is essential to continuously adapt to changes in cognitive health and relational dynamics as the condition progresses [9]. This also applies to changes in communication and social interaction, which can be impaired in individuals with MCI and may affect their ability to participate socially and maintain relationships [10]. Given that cognitive decline in individuals with MCI can negatively impact their relationships with caregivers and potentially lead to interpersonal tensions [11], further research is needed to explore the positive effects of dyadic tablet-based interventions on individuals with MCI and their caregivers. In addition, involving both patients and caregivers in the design of digital applications is crucial for the early identification of design and usability challenges in technology intended for individuals with cognitive impairments [12].

### Study Objectives and Research Questions

We designed this study based on the findings from an earlier investigation by Klein et al [13], in which a prototype of the intervention for cognitive and motor activation, as well as stimulation of social interaction, was tested with individuals with MCI. Insights from Klein et al [13] informed the further development of the touch-based multimedia tablet intervention, incorporating user feedback and refinements to enhance its usability and effectiveness.

Building on previous research, we introduced a dyadic approach involving individuals with MCI and their caregivers to examine how shared tablet-based interventions affect their interaction. By integrating elements of physical, social, and cognitive activation within the touch-based demonstrator, we sought to explore the feasibility of the intervention and the effects experienced by individuals with MCI and their caregivers with regard to their interaction and relationship.

In this study, we addressed the following research questions:

1. Caregiver-patient relationship—How does the use of the intervention influence the relationship between individuals with MCI and their caregivers from their perspectives? What relational dynamics become visible during its use, and how can the intervention be leveraged to foster positive interactions and strengthen the caregiver-patient bond?
2. Feasibility—To what extent is the implementation of a touch-based multimedia tablet intervention in a home environment practicable for individuals with MCI and their caregivers? What challenges and facilitators do they experience during its use?

## Methods

### Ethical Considerations

Following a thorough review process, this study received approval from the Ethics Committee of the University of Furtwangen (application number 24-052). It complies with the tenets of the Declaration of Helsinki. In accordance with institutional guidelines and ethical standards, all participants were assessed for their capacity to provide informed consent before enrollment. A letter containing pertinent information in an easily understandable form and a consent form were distributed to the participants in advance. Only individuals with MCI were included in the study; those with moderate or advanced dementia were excluded to ensure the ability of participants to provide informed and voluntary participation.

The informed consent process was conducted with both the person with MCI and their caregiver present. This arrangement allowed for clarification of the study aims and procedures, and provided an additional opportunity to confirm the participant’s comprehension and willingness to participate. Throughout the testing sessions, both members of the dyad were present, ensuring that the person with MCI was always accompanied by a familiar individual to promote comfort and emotional support during the intervention.

All participants were anonymized. No financial compensation was given to the participants.

### Overview of This Study’s Design

**Figure 1:**
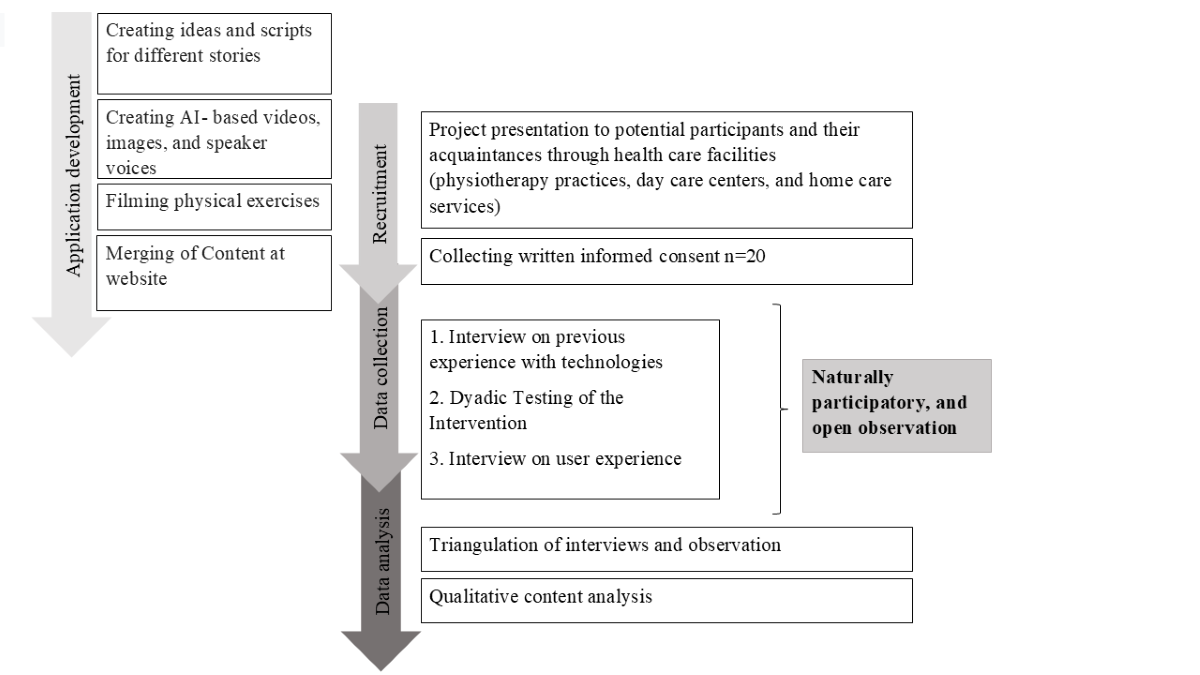
Application development and study procedure. AI: artificial intelligence.

We used a qualitative triangulation approach, combining 20 naturalistic observations of participants and 20 interviews, with data from each method systematically aligned. The research was conducted in Germany and the reporting adheres to the Standards for Reporting Qualitative Research [14]. A summary of the application development and the study procedure is provided in Figure 1.

### Intervention Development Process

We drew upon the TiDIER (Template for Intervention Description and Replication) [15] checklist as a guiding framework to structure the description of the intervention (Table 1). This checklist provides a structured approach to outlining how interventions are designed, delivered, and evaluated, supporting transparency, reproducibility, and adaptability. Using these guidelines as a reference, we aimed to enhance the clarity and rigor of the intervention’s description while allowing for flexibility in its adaptation to the specific research context. The intervention was collaboratively designed and developed by a multidisciplinary research team with expertise in health, computer science, and professional health care (including occupational therapists and physical therapists). This approach ensured that both technical and therapeutic aspects were thoroughly considered, resulting in a user-centered, holistic solution.

**Table 1:** Template for Intervention Description and Replication (TiDIER)–based description of the intervention.

Item	Description of item
Why (rationale)	The intervention aimed to improve cognitive, physical, and social functioning in older adults with MCI^a^ and their caregivers. This may include family members, friends, or professional care staff with whom these people with MCI interact on a regular basis.
What (materials and procedures)	A tablet-based digital intervention was developed using a modular design for ease of use. The overarching design principle of simplicity in implementation was adopted to ensure accessibility and ease of use. To this end, a dedicated web platform was developed to support the design and delivery of the tablet intervention. This platform functioned as a modular toolkit, empowering researchers to assemble the stories by combining various media elements, including self-created recordings (images, sounds, and videos); royalty-free images; and sound files. In addition, AI^b^-generated avatars were facilitated through HeyGen and AI-generated speech through ElevenLabs. The interventions were administered through the website as well. The intervention involved the use of a tablet computer (Galaxy Tab A9+), which was provided by the study team. The tablet contained the demonstration tool. An introductory module was included to explain the website's operation and the nature of the tasks (cognitive, physical, and interactive), ensuring caregivers could effectively facilitate intervention. Participants then selected 1 of 4 stories to engage with. These stories included a city trip to Berlin, a visit to a farm, a trip to Spain, and a day in the forest. The explicit design of these stories aimed to address three core task areas: cognitive exercises with a focus on ADLs^c^—these exercises are designed to stimulate memory, attention, and problem-solving skills while maintaining a practical focus on ADL [5]. Examples include planning a travel day, shopping for groceries, and managing the process of paying (examples of the task types can be found in Multimedia Appendix 1). physical exercises related to ADL [6]—these exercises are carefully designed to promote movement and enhance functional independence. Examples include activities such as simulated stair climbing, exercises involving hand movement exercises, and relaxation exercises. These exercises are adapted to the participant’s fitness level and performed either while seated or while standing. interaction exercises [7]—the objective of these exercises is to promote social engagement and the exchange of personal experiences, thereby fostering the relationship between people with MCI and their caregivers. These interactions are structured to incorporate biographical references, enabling participants to share meaningful memories and build connections during the intervention. The selection of exercises and task areas was based on expert consensus [16] and interdisciplinary team input. The chosen story was played through once. Following the story, feedback questions with predefined answer options appeared on the screen, which the participants responded to.
Who provided (intervention delivery)	The intervention was designed to be facilitated jointly by a person with MCI and their caregiver at home. The digital application guided them through the story-based sessions and interactive elements. Before implementation, participants received a structured introduction and training session to familiarize them with the application and its components, ensuring that they felt confident in using the intervention autonomously at home.
How (mode of delivery)	The intervention was delivered through a tablet-based application, designed for independent, dyadic use at home. The tasks and stories were experienced via touchscreen interaction and audiovisual playback.
Where, when, and how much (setting and dose)	The intervention was designed to be used in a home environment or any familiar setting of the participant’s choice. Each session involved playing one story and engaging in associated tasks, with a total intervention duration of approximately 15 to 20 minutes. The intervention was applied in a single session, as this was a demonstration version. For future implementation, regular joint use on a weekly basis is recommended to support continuity, strengthen dyadic interaction, and enhance potential therapeutic effects over time.
Tailoring (customization)	. Participants could choose between 4 stories and 2 levels of difficulty. Within each story, chapters could be skipped or selected freely to adjust the experience to individual preferences and needs. In addition, each story included movement exercises that could be performed at different levels of difficulty, either in a seated or standing position, to accommodate individual physical abilities and safety considerations.
Modifications	No modifications were made during the delivery of the intervention.

^a^MCI: mild cognitive impairment.

^b^AI: artificial intelligence.

^c^ADL: activities of daily living.

### Participant Selection and Recruitment Process

We conducted the intervention in German to ensure linguistic and cultural relevance, as the study took place in rural areas of southern Germany. Recruitment was conducted using a multipronged, community-based approach designed to ensure low-threshold access and preserve voluntariness throughout the process. We initially contacted local institutions (eg, day care centers and physiotherapy practices) via email to introduce the study. After establishing contact, we visited selected facilities in person to present the project to staff and—in the case of day care programs—directly to older adults and their caregivers during regular group sessions. Recruitment in these settings always included an open invitation format, allowing participants to approach us voluntarily after the presentations for further questions or to express interest.

In addition, we actively recruited in dementia cafés and volunteer services. In the cafés, short presentations with a few Microsoft PowerPoint slides were integrated as program points into the regular schedule, usually in informal settings, such as coffee and cake gatherings. These events often attracted individuals with MCI and their family members together, creating a familiar and trusting atmosphere. We demonstrated small elements of the intervention to make it more tangible and understandable, encouraging attendees to decide freely whether they would like to participate. Flyers were distributed to supplement the verbal information.

Interested individuals could then approach us directly and schedule an individual appointment for testing the intervention. The timing and location of these sessions were arranged entirely based on the preferences and availability of both the person with MCI and the caregiver. This flexible and person-centered approach helped build trust and facilitated participation.

The study included participants aged 65 to 95 years with MCI, along with their caregivers. The term “caregiver” was defined as an individual who maintains a close and ongoing relationship with the person with MCI and provides assistance in daily activities, independent of their participation in the study. This could include spouses, family members, caregivers from day care facilities, friends, volunteers, or other relevant individuals who are regularly involved in the care and support of the person with MCI.

Individuals were excluded from participation if they (1) had severe cognitive impairment and were unable to provide informed consent; (2) were diagnosed with delirium, schizophrenia, or depression; (3) had a high risk of falling or significant mobility limitations; and (4) were living in residential care facilities.

Participants were recruited via local health care providers, home care services, and day care centers to ensure sample diversity. Dyadic appointments were arranged by personally contacting potential participants.

### Data Collection

The data collection process was executed during the period spanning from September 2024 to November 2024. A total of 20 dyads participated in testing the tablet-based demonstrator and providing semistructured dyadic interviews [17].

The interviews were conducted either at participants’ homes or during their time in day care and in accordance with a pre-established, semistructured interview guideline (Multimedia Appendix 2), which provided a framework of deductive categories. The guideline was developed by the interdisciplinary research team and was informed by the overarching research questions, previous research on dyadic technology use in dementia care, and the team’s experience with related qualitative studies. It aimed to explore participants’ previous digital experiences; their handling of the intervention; perceived usability and usefulness of the different task types (cognitive, physical, and interactive); and any observed or experienced relational effects. This structure allowed for open-ended responses and follow-up questions based on participant input.

The first part of the interview focused on collecting demographic and personal data, including gender; age; ownership; use; and knowledge of devices such as tablets, PCs, and smartphones. After a brief introduction to the intervention, the caregiver and the respondent tested the tablet-based intervention together. As the last step, a second short interview took place to evaluate and discuss the feasibility of the tablet-based intervention. In addition, participants were asked about their perceptions of one another during the use of the intervention. The interviews were conducted in accordance with a pre-established interview guideline (Multimedia Appendix 2), which provided a structured framework of deductive categories. This process was recorded by audio recordings and naturalistic, participatory, and open observations, and took from 30 to 60 minutes for each dyad. Relevant interview topics were as follows: previous experience with digital technology, user skills, and personal characteristics of dyads; evaluation of tablet use; overall evaluation of the content; evaluation of different task types (physical, cognitive, and social); and shared use and influence on the relationship.

Furthermore, a naturalistic, participatory, and open observation was accomplished by 2 researchers, following an observation protocol that was created based on the deductive categories [18]. During the intervention sessions, one researcher assumed a dual role: one researcher facilitated the session by providing support to the dyads as needed (eg, with technical questions regarding the tablet application) and simultaneously took part in observing the interactions. The second researcher adopted a nonparticipant observer role, sitting slightly apart from the dyad and focusing exclusively on the documentation of field notes using a structured observation protocol.

The observation protocol was developed by the interdisciplinary study team based on previous experience in qualitative feasibility studies and informed by the relevant literature on dyadic interaction and intervention research in dementia and MCI contexts. The guiding questions served to focus the observations on key domains of interest, such as task feasibility, motivation, and dyadic communication, without applying standardized rating scales.

In the observation protocol, the place, time, and sequence of the observed processes were recorded. Also, it was distinguished between observations, contextual information, methodological and role reflections, and theoretical reflections. This distinction ensures that the observations could later be interpreted as such without being prematurely shaped by hasty theorizing [18]. The protocol supported a systematic but open-ended documentation of relevant behaviors, reactions, and interaction patterns, which were later analyzed qualitatively. Specifically, it consisted of 3 parts (Multimedia Appendix 3). One part of the observation protocol was dedicated to the observation of the care dyad. A second part focused on the observation of the caregiver, while the third part entailed the observation of the communication dynamics within the dyad. For better comparability and assignability, guiding questions for each observation category were created. Parts 1 and 2 contained identical topics and guiding questions, whereas part 3 contained other topics that aimed to provide a comprehensive description of the communication dynamics within the dyad. The relevant topics and observation criteria are listed in Textbox 1.

**Figure 2:**
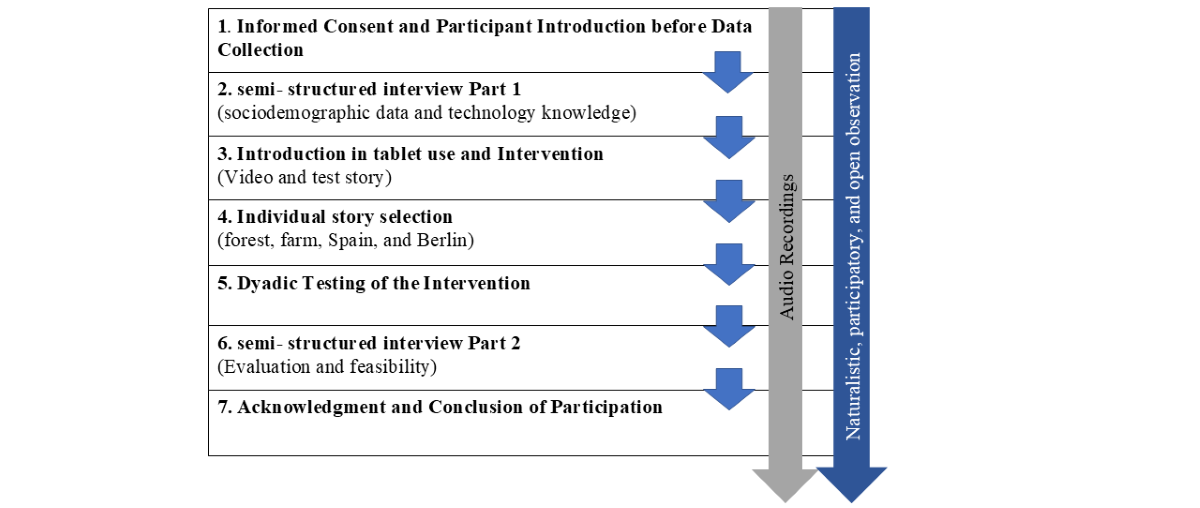
Intervention and data collection process.

The data collection process is summarized in Figure 2.

**Relevant categories and guiding questions of the observation protocol.**

1. Person with MCI (overall feasibility, cognitive tasks, physical tasks, and interaction tasks). For example, how was the motivation during the exercise?
2. Caregiver (overall feasibility, cognitive tasks, physical tasks, and interaction tasks). For example, how was the reaction to the exercise? (positive or negative)
3. Care dyad: communication dynamics and relationship within the dyad (communication, mood, and change in mood; support; attitude of caregivers toward tablet computer; shared use; stimulus for conversation; and overall impression). For example, did the mood undergo any changes? To what extent did the caregiver’s support meet the needs of the patient, and how well was it accepted?

### Data Analysis

The methodological approach followed a systematic and rigorous process to ensure the reliability and validity of the qualitative analysis. The first step involved the creation of a deductive category system based on the overarching themes of the research questions, which provided a structured framework for the analysis, along with a detailed coding guide to ensure consistency during the coding process. Audio recordings of interviews and observations were transcribed. The structured field notes during the observation underwent a double-validation process, whereby 2 researchers independently reviewed the observation data from each other and resolved any discrepancies through discussion. Following this, triangulation of the transcribed interview data and the validated observation protocols was carried out to align insights from multiple sources and enhance the depth of the analysis. The combination of methods allowed us to create results from different perspectives, particularly regarding relationship dynamics. The observations made it possible to include nonverbal communication patterns, power symmetries or asymmetries, and subtle interactions into the analysis.

The qualitative content analysis was conducted based on the methodological approach described by Kuckartz [19] and followed a structured, multistep process. After transcription, both coders independently conducted the initial coding of the interview and observation data using the MAXQDA software (Verbi GmbH), using a combination of deductive categories based on the research questions and inductively emerging subcategories from the material. Theme development was conducted collaboratively. The 2 coders jointly identified and refined preliminary themes through iterative discussions, comparing and synthesizing their interpretations. To ensure analytical rigor and increase trustworthiness, these themes were subsequently reviewed and discussed with additional members of the research team. Discrepancies were resolved through consensus, and categories were adjusted where necessary to better reflect the data. This reflexive and team-based approach aimed to ensure consistency and credibility throughout the analysis process.

Anchor quotations representative of key themes and findings were also identified to illustrate the findings.

### Researcher Characteristics and Reflexivity

In line with the COREQ (Consolidated Criteria for Reporting Qualitative Research) criteria [20], the research team’s backgrounds and positions in the field were considered throughout the research process to enhance transparency and reflexivity. The interdisciplinary study team consisted of health and social scientists with academic training and professional experience in gerontology, rehabilitation sciences, nursing, and qualitative research methods. All researchers had previous experience conducting studies with older adults, including individuals with cognitive impairments and their caregivers. They were trained in qualitative interviewing and observational techniques and were familiar with the ethical and communicative considerations required when working with cognitively vulnerable populations. To ensure reflexivity and reduce potential bias, the research team engaged in regular debriefing sessions in which preliminary impressions and interpretations were discussed throughout the data collection and analysis process. Throughout all interactions, researchers maintained a respectful, nondirective stance to minimize influence on participants’ behavior and responses.

## Results

### Overview

Overall, we recruited 40 individuals, including persons with MCI (n=20, 50%) and their caregivers (n=20, 50%), to participate in our study. The level of experience in using technical devices was grouped into 4 categories: minimal experience, basic experience, moderate experience, and advanced experience. The description of the variables is provided in Table 2.

**Table 2:** Description of the participants’ characteristics.

Variables			People with MCI^a^ (n=20)		Caregiver (n=20)
Age (y), mean (SD)			80.95 (8.27)		60.63 (18.1)
Sex (female), n (%)			15 (75)		16 (80)
**Previous technical experience, n (%)**					
	Minimal experience^b^	7 (35)		2 (10)	
	Basic experience^c^	7 (35)		2 (10)	
	Moderate experience^d^	2 (10)		1 (5)	
	Advanced experience^e^	4 (20)		15 (75)	
**Relationship, n (%)**					
	Spouse	—^f^		5 (25)	
	Child	—		4 (20)	
	Caregiver of a person attending a day care center	—		4 (20)	
	Friend	—		3 (15)	
	Volunteer	—		3 (15)	
	Other	—		1 (5)	

^a^MCI: mild cognitive impairment.

^b^Minimal experience: use of simple, traditional technologies (eg, radio, television, and telephone) and have little or no experience with modern technology (eg, tablets and smartphones).

^c^Basic experience: limited access to or occasional use of modern technology; may own devices (eg, cell phone and tablet) but rarely use them or have had difficulty using them.

^d^Moderate experience: regular use of modern devices, but mainly for basic applications or with support from others.

^e^Advanced experience: in-depth experience and independent, either through intensive previous use or current skills; varied use of modern technology.

^f^Not applicable.

The results were organized along the 2 overarching research questions. On the basis of the qualitative data analysis, 5 main categories were developed deductively and supplemented by inductively derived subcategories. To enhance clarity and traceability, these categories were assigned to the respective research questions according to their thematic relevance:

1. Caregiver-patient relationship—relational dynamics and the influence of the intervention

1.1 Dyadic interaction and support

1.2 Impact of the intervention on communication and engagement

2. Feasibility—practicability, challenges, and conditions for successful implementation

2.1 Personal characteristics and experiences with digital technology

2.2 Feasibility, media, and task engagement

2.3 Future use and recommendations for the intervention

A visualization of this structure is provided in Table 3 to support the reader in contextualizing the categories within the scope of the research questions.

**Table 3:** Deductively developed main categories in relation to superordinate research questions.

Categories	Research question 1: caregiver-patient relationship	Research question 2: feasibility
1.1 Dyadic interaction and support	✓	—^a^
1.2. Impact of the intervention on communication and engagement,	✓	—
2.1 Personal characteristics and experiences with digital technology	Indirectly relevant	✓
2.2 Feasibility, media, and task engagement	—	✓
2.3 Future use and recommendations for the intervention	Implicit, relationship perspective	✓

^a^Not applicable.

### Research Question 1: Caregiver-Patient Relationship—Relational Dynamics and the Influence of the Intervention

#### Dyadic Interaction and Support

The dynamics within dyads were characterized by mutual trust, encouragement, and humor, fostering a positive and engaged atmosphere. Praise and nonverbal reassurance, such as eye contact, helped maintain concentration:

Very good! Where did you learn that? So on with the story.

Caregiver 4

While most dyads communicated harmoniously, differences in communication styles emerged, particularly among married couples, where disagreements over pacing were observed. Some dyads adapted over time, shifting from skepticism to more relaxed interactions. Decision-making varied, with some caregivers assuming control (eg, “don’t keep pressing, I have to read first” [person with MCI 4]), while others facilitated joint decision-making, promoting autonomy. A key aspect was “getting to know each other better” through biographical storytelling (eg, “talking about Berlin or the farm—I think it’s great that you can talk about things you’ve experienced yourself” [caregiver 13]), shared memories, and collaborative tasks. Many dyads perceived the joint implementation as enriching (eg, “yes, that’s good. Two is always better than one [...] and one person can help the other a bit” [caregiver 4]). The medium itself was secondary to the interaction it facilitated, whether through a tablet or other activities. However, challenges such as impatience, paternalism, physical differences, or differing expectations could lead to tension.

Caregivers played a crucial role in technical and content-related support, adapting their assistance based on the person with MCI’s abilities. Some caregivers encouraged independence:

Or do you want to press? With your finger on it.

Caregiver 14

Other caregivers guided tablet use through instruction:

Exactly. Most of the time you have to use the fingertip.

Caregiver 1

As familiarity increased, caregiver intervention decreased, allowing for greater autonomy. However, excessive assistance risked creating dependency, limiting engagement:

Caregiver 5: “If you could choose now, where would you rather go?”

Person with MCI 5: “Spain.”

Caregiver 5: “Yeah. If you want, you can get up. But I don’t care. It’s up to you.”

Content-related support varied from cooperative problem-solving to task takeover. Some caregivers dominated tasks, limiting opportunities for independent learning (eg, “that brings us to 42” [caregiver 13]), while others facilitated responses through guidance and rephrasing. When caregivers provided encouragement and structured support, participation and self-confidence improved. Joint problem-solving fostered a sense of security and trust, allowing individuals with MCI to engage more actively:

You see, it’s very important that you’re there.

Person with MCI 2

#### Impact of the Intervention on Communication and Engagement

The intervention stimulated conversation, particularly through biographical topics and open-ended questions. References to personal experiences (eg, travel, markets, and gardens) encouraged storytelling, deep discussions, and caregiver engagement:

She [Avatar] asked what your best trip was? Do you want to tell?

Caregiver 13

Collaborative exercises enriched interactions, alternating between independent efforts and joint decision-making. Participants valued this approach, stating the following:

Oh yes, but rather together than alone.

Caregiver 12

It gives more motivation, and when you do it as a pair, where both can give answers, that’s nice.

Person with MCI 10

Laughter and shared experiences enhanced motivation, yet caregiver dominance sometimes led to answer anticipation, impacting engagement. Differences of opinion occasionally arose but were mitigated by the structured research setting. While knowledge-based questions occasionally led to uncertainty and insecurity, the intervention proved effective in fostering dynamic exchanges, particularly when topics were personally relevant. In addition to self-reported experiences, observations during the joint use of the intervention revealed moments of closeness and shared engagement. These appeared socially meaningful, particularly when task complexity, autonomy, and role distribution were attuned to the dyad’s needs.

### Research Question 2: Feasibility—Practicability, Challenges, and Conditions for Successful Implementation

#### Personal Characteristics and Experiences With Digital Technology

##### Overview

Interview and observation analyses revealed that the personal backgrounds of individuals with MCI—shaped by their life experiences, social environments, and daily routines—strongly influenced their perspectives and engagement. Key themes emerged, including regional origins, cultural influences, former professions, and family roles. For example, one participant described how her career as a seamstress and raising her children structured her daily life. Health limitations, such as visual or hearing impairments, as well as technological barriers, also impacted their independence and activities.

Family support played a crucial role in overcoming daily technological challenges. One participant shared how her daughter introduced her to mobile phones:

Yes, and when my daughter got a new cell phone, she always gave me her old one and always said, do it, and I’ve been doing it for a few years. My grandson always helped me at the beginning when he was there, yes, then we write emails to each other and try to do something like that.

Person with MCI 17

Participants’ experiences with technology varied widely, from complete unfamiliarity to routine use. Personal attitudes and biographical contexts influenced these differences. Some participants expressed skepticism, rejecting digital devices as unnecessary:

Well, I’m definitely not a computer freak.

Person with MCI 8

I have my TV, that’s enough.

Person with MCI 11

However, others had extensive experience and integrated technology into their daily lives:

I did all my written work online. And then I have six grandchildren of all ages. And they keep me fit.

Person with MCI 9

Professional and family contexts played a crucial role in the development of technical skills.

##### Acceptance and Rejection of the Technology

Survey responses showed a complex picture of both acceptance and skepticism regarding the intervention. Some participants were enthusiastic, viewing the technology as stimulating and beneficial (eg, one participant stated that they “absolutely wanted to take part in the study” [person with MCI 17]). Key factors for acceptance included feelings of success, motivation, and interactive elements. Two participants were motivated by the desire to promote their own health through the movement exercises (eg, “it’s healthy” [person with MCI 5] and “movement promotes blood circulation, and blood circulation in turn promotes the ability to think” [person with MCI 20]). Participants also highlighted the value of memory activation and cognitive stimulation:

It was fun to name the things that you know. Yes, then you’re happy that you already know something and that you’re somehow making progress.

Person with MCI 17

I’m interested in something like that, so that it sticks in my memory. I have a problem with my memory because the doctor said it wasn’t very good.

Person with MCI 18

The social aspect contributed positively to acceptance, with some participants emphasizing that technology facilitated shared experiences:

It was nice to do it together.

Caregiver 6

However, technical challenges and design limitations posed barriers to acceptance. Some participants were skeptical about the relevance of the technology in their daily lives:

Do you really need it?

Caregiver 1

The success of the intervention depended on intuitive usability, cognitive stimulation, and interactive design, while obstacles included technical difficulties, content adaptation, and perceived relevance. In summary, acceptance was driven by positive user experiences, while rejection stemmed from usability concerns and doubts about the technology’s practical value.

##### Content Aspects and Choice of Stories

Participants’ topic preferences were strongly influenced by personal interests and past experiences. The “forest story” was the most frequently chosen, as the forest is a familiar and meaningful place for many. Familiarity with a topic often led participants to engage more, as they associated it with positive past experiences, such as family vacations. Conversely, a lack of personal connection acted as a barrier, leading some to reject certain topics:

Spain, that’s not for us, we’ve never been there.

Person with MCI 3

However, for others, unfamiliar topics sparked curiosity and provided an opportunity to learn something new. The forest theme received broad acceptance and positive responses due to personal connections with nature:

We lived in the Black Forest for many years and spent a lot of time in the forest, and we still talk about those beautiful walks today.

Person with MCI 3

I am a nature lover through and through.

Person with MCI 17

Many recalled special memories, such as hiking, animal-watching, or past experiences in the military, particularly from a time when they were not yet physically restricted. The farm story also resonated well with participants, as it was both biographically relevant and generally familiar. Many had childhood memories or past visits to farms, while others found the rural lifestyle intriguing:

We have four sons, and when they were relatively young, we always spent our vacations on a farm.

Caregiver 9

The Spain story provided various points of reference, particularly for those with travel experiences or a general interest in the region:

I have already driven through Spain. As I said, above Coimbra, to Portugal, to Fatima, and then back via Madrid and back to Germany.

Person with MCI 20

The Spain story was often described as informative and entertaining, although some found it more challenging than other stories. The Berlin story triggered reflections on the city’s history, especially regarding the Berlin Wall and the divided past of the city:

Berlin is beautiful, big, loud, all sorts of things, but um, the wall, it makes you thoughtful.

Person with MCI 13

Participants’ interaction with the buttons varied widely. While some tapped independently, others were hesitant, required assistance, or sought clarification. The floating “Next” button caused occasional confusion, as its function was not immediately clear. Similarly, uncertainty arose with selection buttons under images, as some participants attempted to tap the images directly, leading to frustration when no response occurred. In addition, prolonged pauses between slides without interactive elements resulted in uncertainty about the next steps, with one participant explicitly asking for guidance:

So where do I press? It’s a bit of uncharted territory, isn’t it? You have to think about it.

Person with MCI 3

At the beginning of cognitive questions, answer options were initially hidden, leading to confusion that diminished once they became visible.

Initial difficulties in using the tablet were common, often due to unfamiliarity with the technology. Typing proved challenging for some, especially those with long fingernails or those who exerted excessive pressure on the touchscreen. While some participants remained hesitant at first, many gained confidence over time and eventually typed independently. Notably, participants with no previous tablet experience were initially skeptical but adapted quickly. One participant described typing as “new territory” but ultimately found it enjoyable. Despite early uncertainties, many reported increasing speed and confidence with continued use.

#### Feasibility, Media, and Task Engagement

The use of rewards, such as animal pictures after task completion, proved to be an effective emotional motivator, fostering joy and enthusiasm in both individuals with MCI and caregivers. Statements such as “once a lamb and once the dog. That builds you up” (person with MCI 2) illustrate how such elements encouraged engagement. The reward system not only increased motivation and attention but also enhanced social interaction within dyads:

Tom (avatar): “Well done. That was correct.”

Caregiver 2: “The contestant has 100 points” (laughs).

Person with MCI 2: “Yes, yes. Now he’s continuing. Look there the donkey said ‘Bravo’ That’s funny.”

Visual media, particularly nature-related images (eg, forest paths, flower meadows, streams, and animals), were consistently well received and described as visually appealing and emotionally uplifting:

They were beautiful, especially the forest and everything.

Person with MCI 1

Videos featuring landscapes and moving animals provided an immersive experience:

Seeing the landscape again, from a different perspective from above.

Caregiver 1

Narrator: “Ella watches as an orange butterfly lands on a blade of grass. To remember the moment, Ella takes another.”

Person with MCI 1: “Oh, that’s a beautiful fellow. I think I’ve barely seen any this year. Oh wait, a yellow one did fly by the other day.”

Artificial intelligence (AI)–generated content (eg, images, speaker voices, and avatars) was widely accepted, seamlessly integrating into participants’ experiences without compromising authenticity:

Interviewer: “Tom—this person—we created him using artificial intelligence. So it’s not a real person, but someone we generated with a computer.”

Caregiver 15: “Oh, but at the time, it wasn’t really noticeable that it wasn’t a real person.”

Computer-generated voices and sound effects were generally perceived as clear and natural, although minor issues such as choppy audio or low volume were noted, but did not significantly affect comprehension. Background sounds, such as birdsong and flowing water, were praised for enhancing the atmosphere. A well-balanced combination of voices and ambient sounds was found to improve the overall user experience. Text-based and interactive elements received mixed feedback. While most found the font legible, some suggested increasing the font size, stating, “slightly larger” (dyad 17). Button placement and touch recognition issues were noted, with recommendations to space buttons further apart for better usability.

The range of tasks was generally well received, particularly movement-based activities, which were seen as beneficial for both physical health and cognitive activation:

Movement promotes blood circulation, which supports thinking.

Person with MCI 20

Personal anecdotes often accompanied exercises, for example, “feeding chickens” (farm story) or “running up the cathedral tower” (Spain story), enhancing engagement. Dyadic interaction influenced task acceptance, while relaxation and breathing exercises elicited mixed reactions—some found them calming, others reacted humorously. Interaction exercises encouraged quick responses and biographical storytelling (eg, farm visits, nature, and markets), effectively activating memories and fostering dialogue. Cognitive tasks were generally perceived as appropriate, although some participants, particularly those with relevant backgrounds, desired greater challenges. Many participants enjoyed problem-solving tasks, expressing enthusiasm through clapping, smiling, or laughing. Open-ended knowledge questions encouraged discussions and thought sharing, making them especially popular. The variety of question types was widely appreciated.

#### Future Use and Recommendations for the Intervention

The tablet-based intervention received positive feedback. One participant noted the following:

Time passed quickly, and it was a nice change.

Caregiver 16

Many participants were open to future use, even those with limited previous tablet experience:

Very good, it was very good. I’m surprised. She [Person with MCI] read and listened to everything, responded, and with such great interest. Better than usual.

Caregiver 18

One participant with MCI 3 expressed confidence:

I think I can do it now...What do you call it? Tablet? That’s easy.

In response, caregiver 5 added, “You can still learn to use a tablet at 92, right?” However, some saw limited personal use, mainly as an activity to share with grandchildren:

If my grandson suggested it, I’d join in.

Caregiver 8

Others viewed it as a one-time experience:

That was nice, but just once.

Person with MCI 15

Some rejected it outright:

I don’t feel like it. Why should I learn this?

Caregiver 5

I have my TV, that’s enough for me.

Person with MCI 1

Overall, willingness to use the intervention was mostly positive, but some participants preferred alternative health-promoting activities over learning new technology. Participants suggested adding more animals and matching sounds to the forest story, making background noise quieter to improve focus, and using more relevant feedback images. They recommended a larger touchscreen with bigger, clearly highlighted buttons and a more intuitive introduction with arrows pointing to where to press. Zooming options for images would help people with vision impairments. Shorter pauses between slides and longer stories (eg, the Berlin story was “too short”) were also preferred. Participants favored focusing on one type of exercise (cognition, interaction, or movement) and wanted more feedback on quiz answers, as well as opportunities for independent writing tasks. A sing-along song for more interactivity was suggested. In terms of content, participants expressed interest in travel stories set in places such as Spain, the Netherlands, or local cities with landmarks, castles, and regional foods. They also wanted stories about trips to the sea, the mountains, the Camino de Santiago, or farm visits. Other ideas included traditional crafts, iconic routes such as Route 66, boat tours, market visits, and exploring local heritage and cuisine.

## Discussion

### Principal Findings

This study explored the feasibility of a dyadic, touch-based multimedia intervention for individuals with MCI and their caregivers in a home setting, focusing on its impact on caregiver-patient relationships and dyadic communication. The intervention, incorporating physical, cognitive, and interactive exercises, aimed to address cognitive and relational challenges through shared activities.

A key finding was the positive influence on caregiver-patient relationships. Shared activities facilitated connection, mutual support, and emotional intimacy, particularly through biographical elements, which sparked meaningful dialogue and strengthened bonds. Humor, encouragement, and collaborative problem-solving enhanced communication. Caregivers played an essential role, with varying levels of involvement. Some encouraged autonomy, while others were more controlling, occasionally limiting independence. Striking a balance in caregiver support is key to maintaining a collaborative dynamic.

The intervention was feasible for home use, with participants adapting well despite initial unfamiliarity. The integration of AI-driven visual and auditory elements was particularly promising, as most participants did not perceive these features as unnatural. However, challenges such as confusion with interactive buttons, slide transition delays, and difficulties for those with limited previous experience pointed to areas for improvement, such as an intuitive interface, larger buttons, and faster system responsiveness. Mixed reactions on future use highlighted a preference for customization. While many participants were enthusiastic about continuing, others preferred traditional activities, emphasizing the need for relevant and varied content. Feedback mechanisms, adaptive difficulty, and offline accessibility could enhance its appeal.

### Comparison With Previous Work

According to our study, a dyadic, touch-based multimedia intervention for individuals with MCI and their caregivers in a home setting is feasible. Participants’ difficulties mainly depended on their previous technical experience, with those having moderate or advanced experience finding the tablet easier to use. Despite initial uncertainties, most individuals with MCI gained confidence over time. An introductory video facilitated familiarization with the tablet, supporting findings by Quintana et al [21]. However, unfamiliarity with touch surfaces and interactive fields created challenges, such as pressing too hard or not knowing where to press. These issues were exacerbated by inconsistent button positioning, suggesting the need for uniformity in future designs. The dyadic concept proved helpful, as caregivers provided support, enhancing interaction and helping overcome difficulties, as noted by Klein et al [13].

User acceptance was high, with participants describing the intervention as both enjoyable and cognitively engaging. Two levels of difficulty were implemented to accommodate varying conditions and daily performance, which the participants appreciated. Our findings support the importance of adapting multimedia applications to users’ needs [16]. However, technical barriers, such as limited Wi-Fi access, posed challenges, leading to disruptions such as audio interruptions. These issues could be mitigated with an offline version, now available, which should be considered for future interventions. The perceived relevance of the technology played a crucial role in acceptance, as users who found the tablet relevant demonstrated higher levels of engagement. This point has been emphasized by Chen et al [7] and Thordardottir et al [22]. These findings can be better understood through the lens of the technology acceptance model [23], which posits that 2 key factors—perceived usefulness and perceived ease of use—determine users’ acceptance of a technology. In our study, both aspects emerged as highly relevant: while many participants appreciated the intervention’s cognitive and relational value (usefulness), others were discouraged by technical hurdles or limited familiarity with touch-based interfaces (ease of use). This aligns with previous findings [24,25] that emphasize the importance of intuitive design and contextual relevance in enhancing user engagement. Integrating the technology acceptance model in future evaluations may provide a more structured framework for understanding acceptance dynamics and guide further refinement of the intervention.

Content relevance also played a role, with participants preferring stories connected to their own experiences, such as those involving forests and farms. City stories were less engaging, which aligns with the findings of Klein et al [13] that content relevance impacts user acceptance. A participatory approach in design could further ensure that the content meets users’ needs, as suggested by Holthe et al [26]. Our results indicate that targeted adaptations, involving both people with MCI and caregivers in the design process, are key for long-term acceptance and success.

The study highlights both positive impacts and challenges for the relationship between individuals with MCI and their caregivers. A key finding is that biographically oriented content can strengthen the caregiver-patient relationship by facilitating personal dialogues and shared reminiscence, which enhances emotional intimacy and mutual understanding. This finding aligns with that of Ryan et al [27], who showed that biographical content strengthens emotional bonds in tablet-based interventions. Similarly, Hoel et al [6] highlighted how technology can enhance social interaction and alleviate communication pressures. Klein et al [13] also noted that caregiver support is crucial for the success of tablet-based interventions.

Caregivers played a central role in guiding and encouraging active participation of individuals with MCI, reinforcing the importance of adaptive support as seen in the study by Ingebrand et al [28]. However, challenges arose when caregivers became overly controlling, limiting the autonomy of individuals with MCI. This finding aligns with that of Ingebrand et al [28], who emphasized the need for a balanced approach to caregiver involvement. Gilson et al [29] similarly pointed out that while technology-based interventions can strengthen emotional bonds, they also risk inducing frustration if caregivers overstep their supportive roles or if the technology is perceived as too complex.

Moreover, our study suggests that the home environment positively influenced the intervention’s success, supporting the findings of Gilson et al [29], who showed that home-based interventions can yield favorable outcomes for individuals with dementia. Future research should focus on investigating long-term relational dynamics, optimizing caregiver involvement, and assessing the scalability of such interventions. Tailoring content to cultural and biographical contexts will be crucial for maintaining relevance and increasing acceptance. Future studies should focus on optimizing caregiver involvement, tailoring content to cultural and biographical contexts, and assessing scalability.

### Strengths and Limitations

We used a qualitative research approach, which is particularly well suited for exploring lived experiences and subjective meanings in complex social contexts [30]. By implementing methodological triangulation, we combined different data sources and perspectives, increasing the robustness and validity of our findings. In addition, the study explicitly addressed the dyad as a unit, acknowledging the dynamic interaction and relational dynamics between individuals with MCI and their caregivers. This holistic approach reflected the real-life caregiving context, ensuring that both social and cognitive aspects were considered in evaluating the intervention’s impact. The study also benefited from a diverse recruitment strategy, including participants from various caregiving settings, such as home care, daycare facilities, and physiotherapy practices, which enhanced the practical relevance of the findings. Furthermore, the integration of observational data alongside interviews provided deeper insights into participants’ actual behavior, reducing the risk of self-report bias.

Despite these strengths, several limitations must be acknowledged. One limitation was the lack of internet access among a substantial portion of the target population, posing challenges as the intervention was exclusively online. However, the dependence on technology may have posed a barrier for individuals less familiar with digital tools. Another potential limitation was social desirability bias, as participants may have provided overly positive feedback during interviews. This risk was mitigated by observations and researcher cross-validation. Furthermore, the study provides a short-term perspective on the intervention, meaning that insights into long-term engagement and effects remain limited. Future research should explore how sustained use influences outcomes over time.

Overall, this study offers valuable insights into dyadic interactions in a digital intervention while highlighting both opportunities and challenges in its implementation. While this feasibility study was not designed to evaluate relational outcomes, the data suggest that shared use of the intervention may open opportunities for meaningful dyadic interaction. Future research should further explore the potential relational impact in a targeted design.

### Conclusions

This study evaluated the feasibility of a dyadic, tablet-based multimedia intervention for individuals with MCI and their caregivers in a home setting, with a focus on its impact on caregiver-patient relationships. The findings indicate that the intervention was feasible, with initial uncertainties mitigated by thematic introductions and caregiver support, even for participants with limited technical experience.

A key factor in user acceptance and feasibility was the relevance of the intervention to participants’ daily lives. One of the study’s strengths was the implementation of 2 difficulty levels, allowing for adaptability to the fluctuating cognitive performance of individuals with MCI. Participants generally provided positive feedback, partly attributing benefits to cognitive and physical engagement. Our findings also underscore both the potential and challenges of using technology to support caregiver-patient relationships. Biographically oriented content appeared to facilitate social interaction and strengthen bonds. Furthermore, the integration of AI-generated visual and auditory elements was well received, as most participants did not perceive them as unnatural. This suggests that AI-based storytelling can enhance engagement and provide more personalized content. In future interventions, AI could be used to generate individualized, person-centered narratives that spark meaningful conversations within the dyad, further strengthening relationships and improving communication.

However, as this was a cross-sectional study, no conclusions about long-term effects can be drawn. Future research should prioritize longitudinal studies to assess sustained relational impacts and explore strategies to optimize caregiver involvement.

## Appendix

Multimedia Appendix 1 Examples of the task types within the demonstrator.

Multimedia Appendix 2 Guidelines for the dyadic interviews—English version.

Multimedia Appendix 3 Observation protocol—English version.

## Abbreviations

ADL: activities of daily living
AI: artificial intelligence
COREQ: Consolidated Criteria for Reporting Qualitative Research
MCI: mild cognitive impairment
TiDIER: Template for Intervention Description and Replication
