# Supplementary material for: Evaluating the Feasibility of a Dyadic, Touch-Based Multimedia Tablet Intervention and Its Effects on the Caregiver-Patient Relationship Among Individuals With Mild Cognitive Impairment: Qualitative Triangulation Study
Source: JMIR Aging. 2025 Aug 28;8:e75189. doi: 10.2196/75189 (PMC12426569; doi:10.2196/75189)
Supplement: Multimedia Appendix 1 [file aging_v8i1e75189_app1.docx]

## Appendix 2: Examples of task types within the demonstrator

1. Cognitive exercises


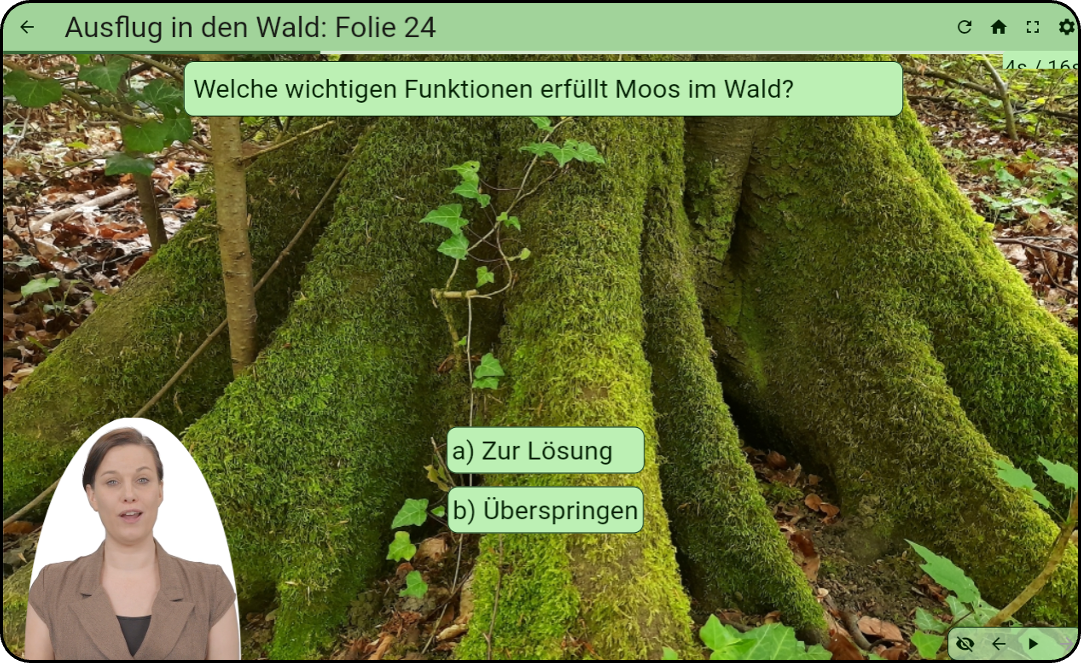


Illustration: Example of cognitive exercises in the forest stroy


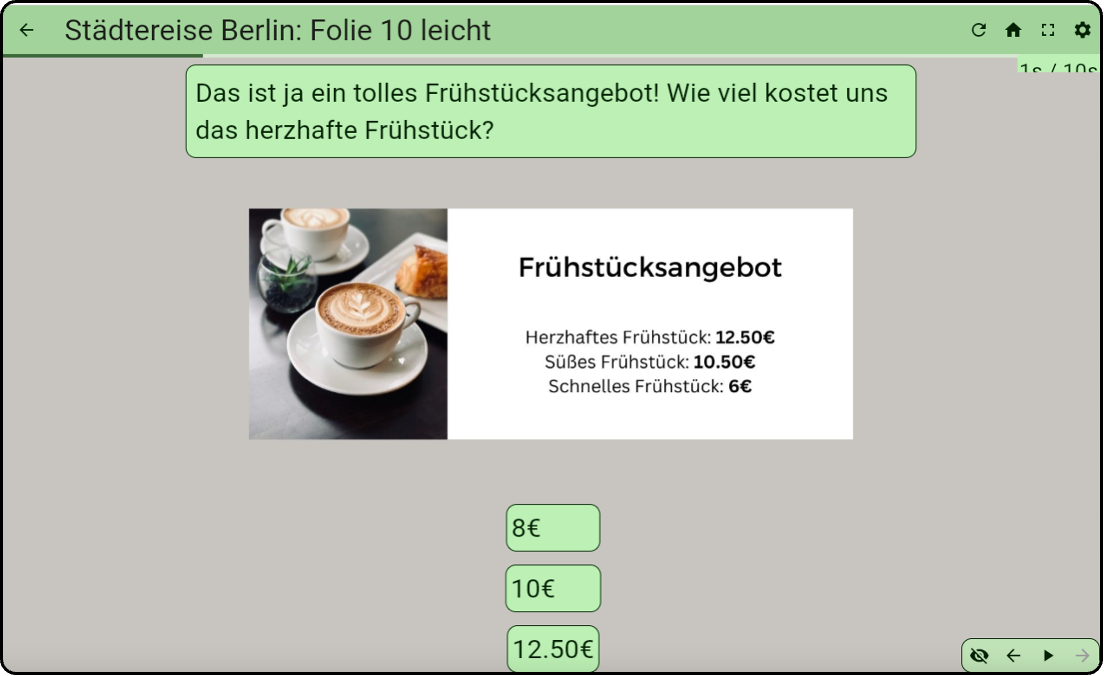


Illustration: Example of cognitive exercises in the Berlin stroy


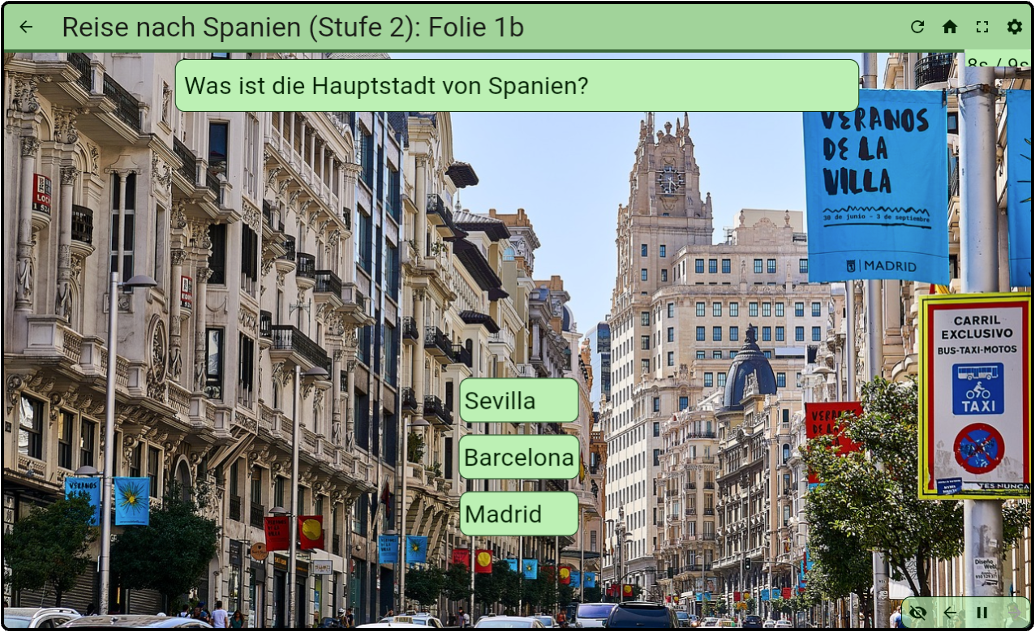


Illustration: Example of cognitive exercises in the Spain stroy


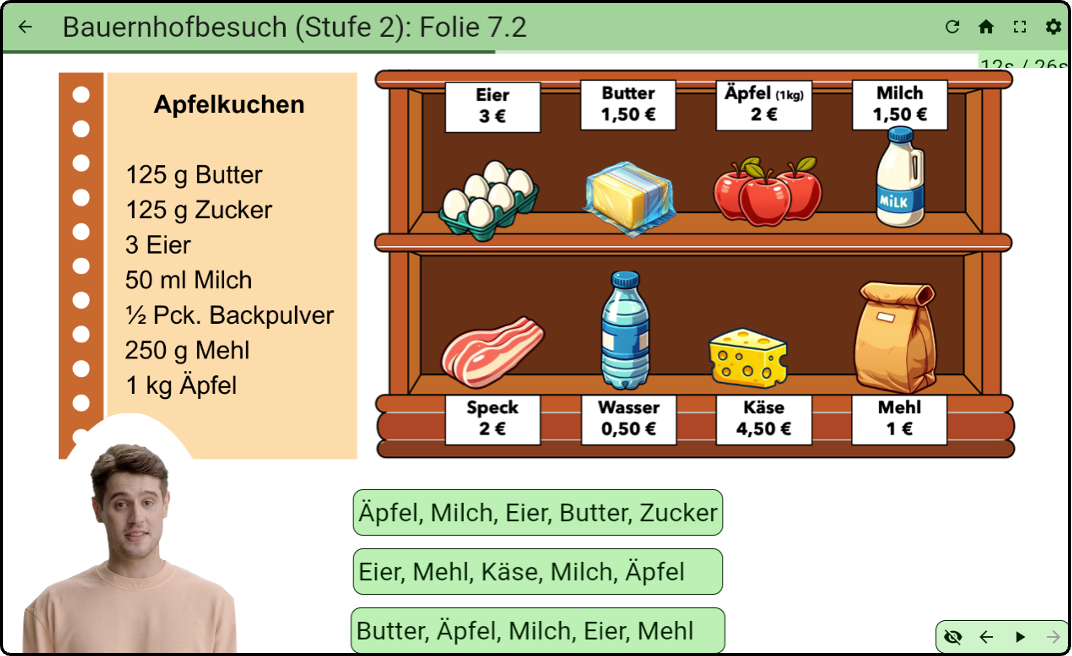


Illustration: Example of cognitive exercises in the farm stroy

1. Physical exercises

**
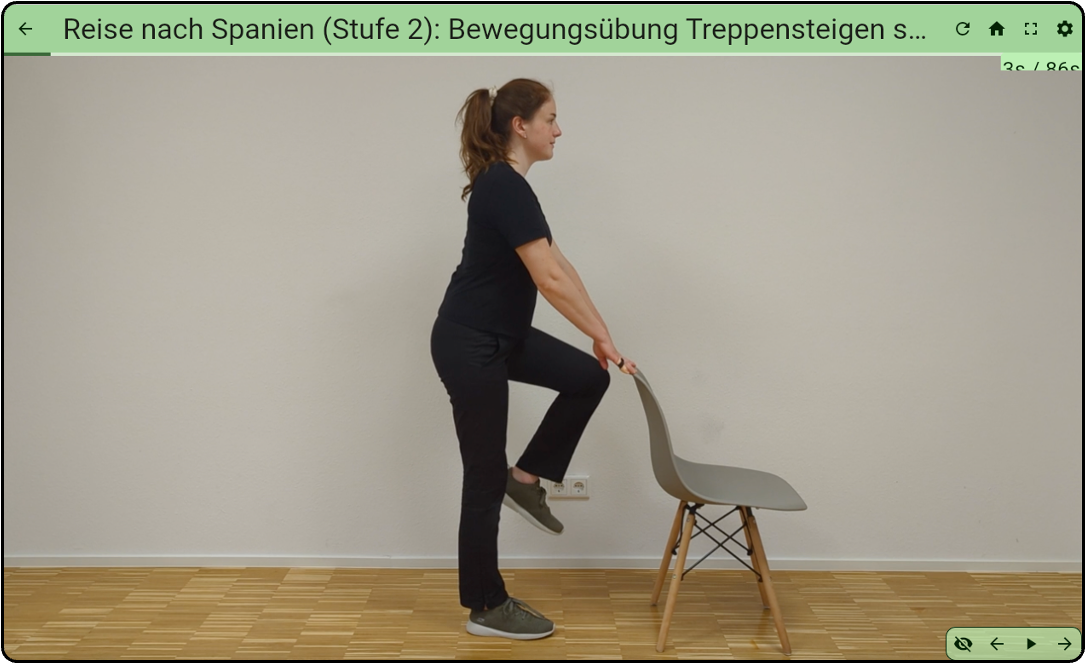
**

Illustration: Stair climbing exercise

**
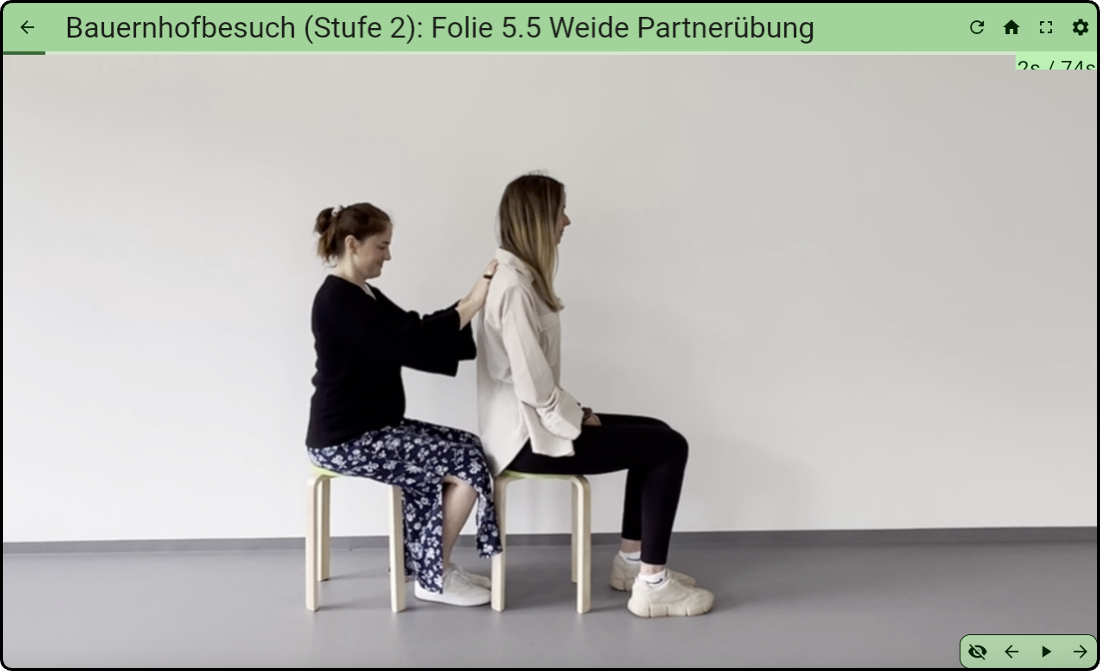
**

Illustration: Movement exercise with a partner

**
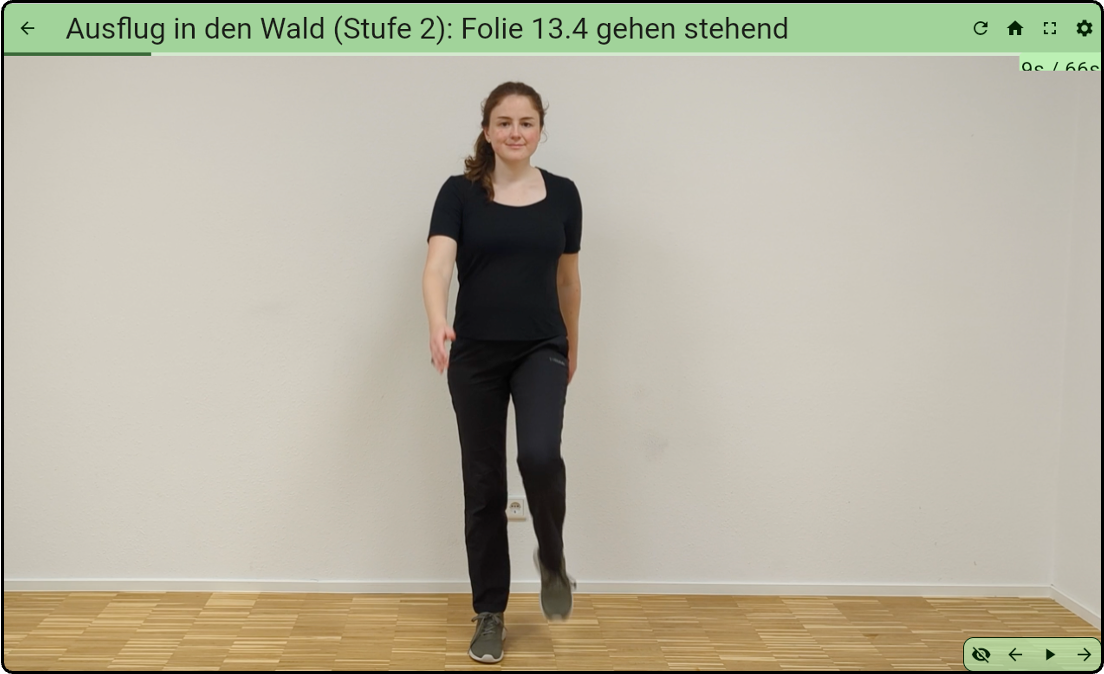
**

Illustration: Walking exercise

**
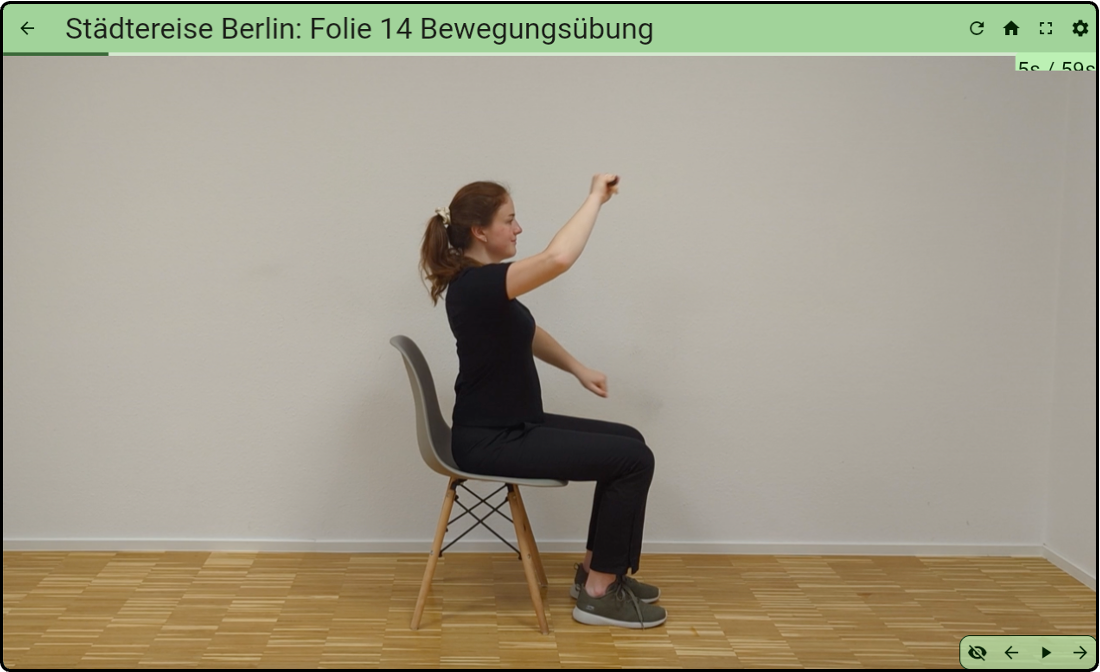
**

Illustration: Cycling exercise

1. Interaction exercises

**
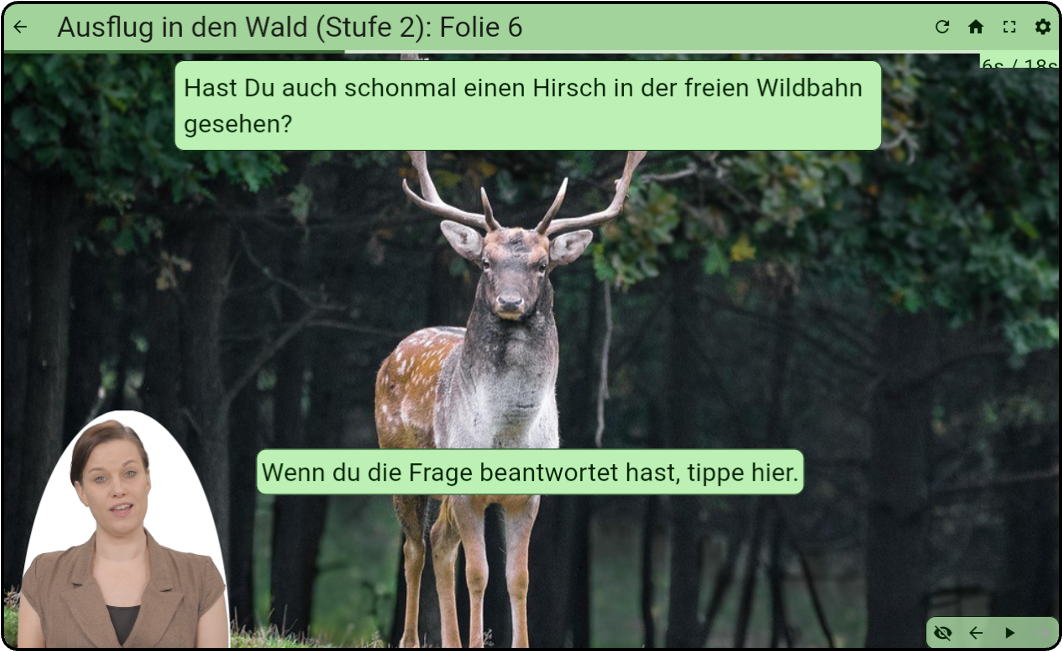
**

Illustration: Exemple of Interaction exercises in the forest story

**
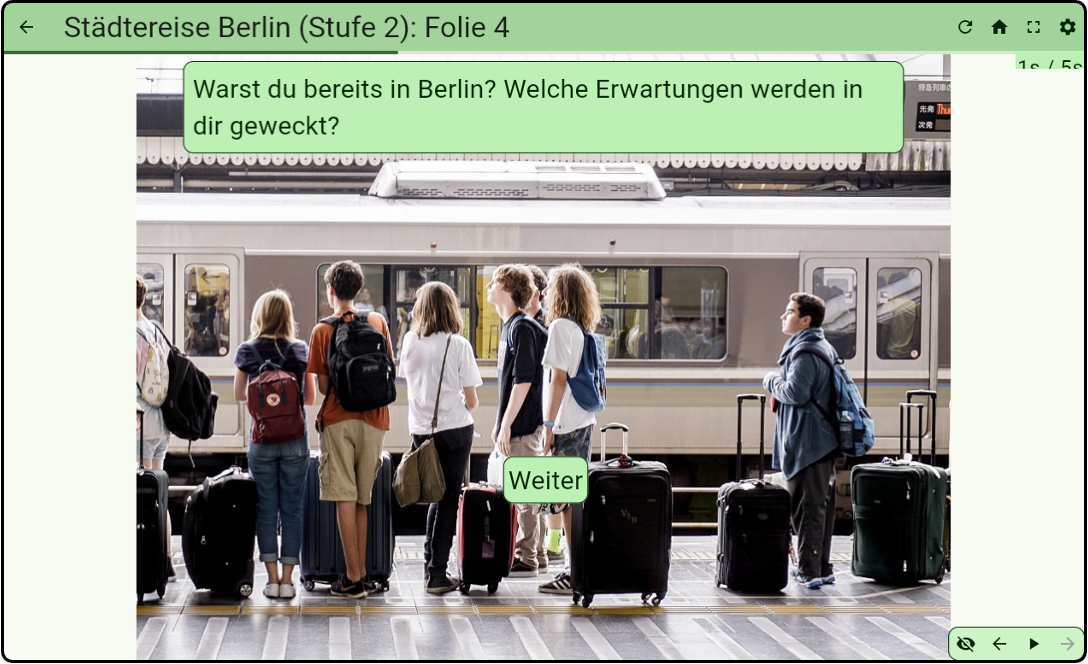
**

Illustration: Exemple of Interaction exercises in the Berlin story

**
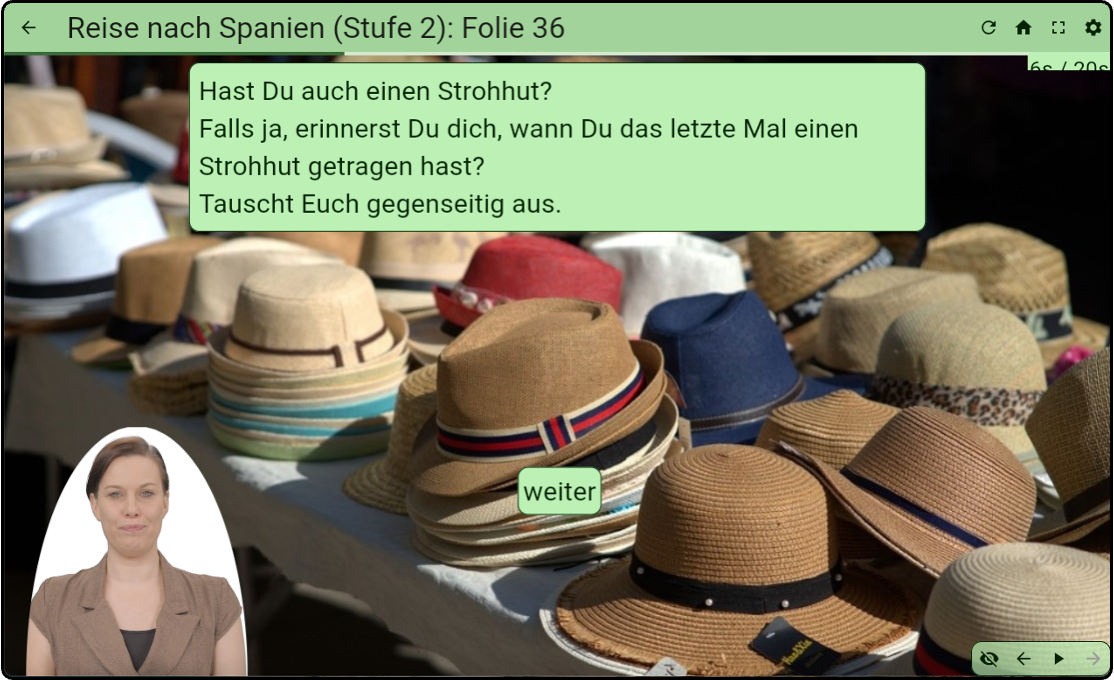
**

Illustration: Exemple of Interaction exercises in the Spain story

**
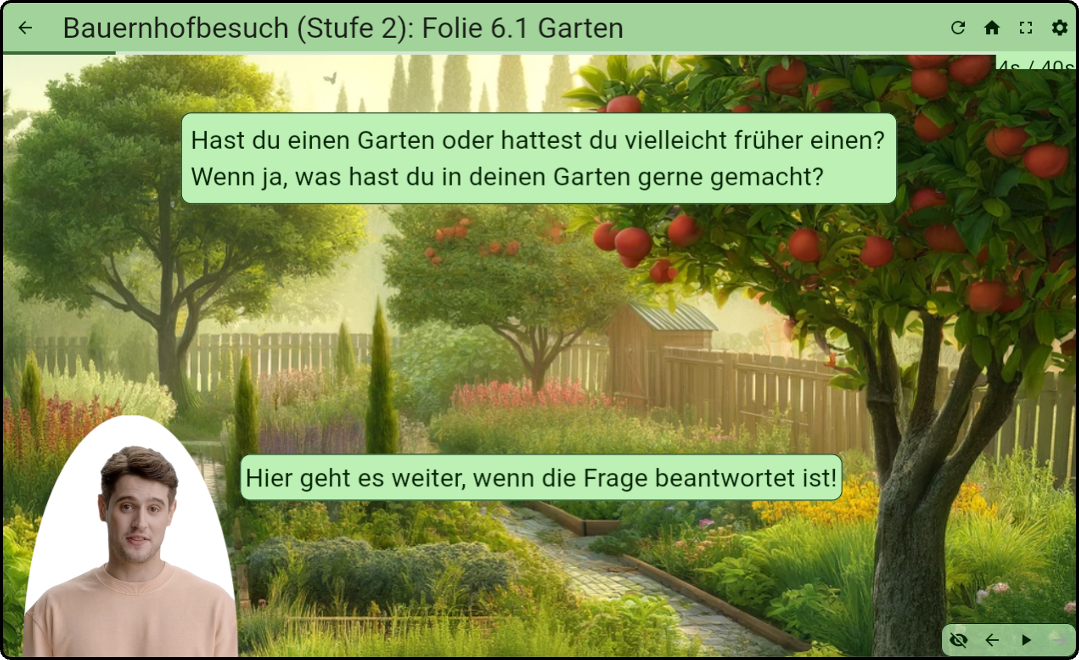
**

Illustration: Exemple of Interaction exercises in the farm story
