# Supplementary material for: Evaluating the Feasibility of a Dyadic, Touch-Based Multimedia Tablet Intervention and Its Effects on the Caregiver-Patient Relationship Among Individuals With Mild Cognitive Impairment: Qualitative Triangulation Study
Source: JMIR Aging. 2025 Aug 28;8:e75189. doi: 10.2196/75189 (PMC12426569; doi:10.2196/75189)
Supplement: Multimedia Appendix 2 [file aging_v8i1e75189_app2.docx]

## Appendix 2: Guidelines for Dyadic Interviews

**Introduction**
Thank you for participating in our project and taking the time for it! Everything you share here and everything we do together will remain confidential. Your data will be anonymized so that no one can later identify who you are. We are recording the conversation with an audio device, as discussed. Our goal is to find out how well a story can be experienced on a tablet together with a familiar person and whether there are any challenges. Some tasks will stimulate thinking, others involve movement. Participation is voluntary, and the fun of the activity is central. Before we start the story on the tablet, we have a few general questions about your previous experience with digital devices. This helps us understand how familiar you are with such technologies.

| **Topic** | **Guiding question / Prompt** | **Relevant content aspects (must be mentioned)** | **Follow-up questions  (if appropriate)** | **Supportive questions** |
| --- | --- | --- | --- | --- |
| **Relationship** | What is your relationship to your reference person? | Relationship to each other (spouse, parent-child, sibling, uncle/aunt, neighbor, etc.) |  |  |
| **Age** | How old are you? |  |  |  |
| **Previous experience / digital competency** | Have you had any experience with a tablet or other electronic devices? | Is the usage independent or with the help of your reference person?  Potential problems with usage | If no: Do you have experience with other electronic devices like mobile phones or computers?  If yes: What experiences have you had using them? | Nonverbal Prompts to Keep the Conversation Going  Can you explain that further? Do you have an example? |
| **…INTERVENTION TESTING…** (Transition to observation guideline) | | | | |
| **AFTER THE INTERVENTION** | | | | |
| **Application** | Were you able to use the application easily, or were there difficulties? | What was particularly easy? What was particularly difficult? | If too easy:  Was it too easy?  If difficulties: Where would you have needed (more) help? | Can you give an example?  How was it with XY? |
| **Stories/Content** | How did you like the stories in the intervention? | Comprehensibility  Duration of the application  Voice (if AI was used) | If criticism: What can be improved? | How did you feel about XY? |
| **Cognitive exercises** | How did you like the thinking exercises? | How was the difficulty level perceived? Was the task clear? | What did you particularly like/dislike? Any additional comments? | Can you elaborate that?  What did you think of XY? |
| **Movement exercises** | How did you like the movement exercises? | Could they be performed easily or were there difficulties? | What did you particularly like/dislike?  Are there any additional comments? |  |
| **Biography exercises** | In some tasks, you were asked personal questions about your past. How did you feel about that? | Was a personal connection possible? Emotions? |  | How did you feel during XY? What did you think about XY? |
| **Images** | How did you like the images in the application? | What did you like or dislike? | If something was disliked: What didn’t you like? What would you prefer? | Do you have a specific image in mind or can you name one?  How did you like XY? |
| **Joint usage** | How did you like the joint usage? | Can you imagine using it in the future outside the study?  Did you enjoy using it?  What was difficult? | If yes: Why?  If no: Why not?    What aspects of the intervention were especially helpful or obstructive (regarding future use or personal value)? | Does XY play a role here? |
| **Impact on the relationship** | ​​ How did you perceive the interaction during the intervention? | Feelings during it  Changes in the relationship compared to usual? | Do you think long-term, joint usage could influence your relationship positively or negatively? | Can you elaborate or give an example?  Does XY play a role here? |
