# Supplementary material for: Evaluating the Feasibility of a Dyadic, Touch-Based Multimedia Tablet Intervention and Its Effects on the Caregiver-Patient Relationship Among Individuals With Mild Cognitive Impairment: Qualitative Triangulation Study
Source: JMIR Aging. 2025 Aug 28;8:e75189. doi: 10.2196/75189 (PMC12426569; doi:10.2196/75189)
Supplement: Multimedia Appendix 3 [file aging_v8i1e75189_app3.docx]

## Appendix 3: observation protocol

| **Pseudonym dyad:** | **Story:** |
| --- | --- |

1. **Testperson**

| **Topic** | Observations, location/time, contextual information, methodological and role reflection, theoretical reflection |
| --- | --- |
| **General eeasibility**   - Difficulties in use (sound, images, text size) - Technical problems - Reactions to the content - Reactions to visual/auditory design - Attitude/reaction to tablet use |  |
| **Cognitive exercises**   - Expressed/perceived difficulties - Difficulty level appropriate - Comprehension issues - How was the exercise received? - Reactions (positive/negative) - Motivation during conduction |  |
| **Movement exercises**   - Expressed/perceived difficulties - Difficulty level appropriate - Comprehension issues - How was the exercise received? - Reactions (positive/negative) - Motivation during conduction |  |
| **Biography exercises**   - Expressed/perceived difficulties - Difficulty level appropriate - Comprehension issues - How was the exercise received? - Reactions (positive/negative) - Motivation during conduction |  |

1. **Caregiver**

| **Topic** | Observations, location/time, contextual information, methodological and role reflection, theoretical reflection |
| --- | --- |
| **General feasibility**   - Difficulties in use (sound, images, text size) - Technical problems - Reactions to the content - Reactions to visual/auditory design - Attitude/reaction to tablet use |  |
| **Cognitive exercises**   - Expressed/perceived difficulties - Difficulty level appropriate - Comprehension issues - How was the exercise received? - Reactions (positive/negative) - Motivation during conduction |  |
| **Movement exercises**   - Expressed/perceived difficulties - Difficulty level appropriate - Comprehension issues - How was the exercise received? - Reactions (positive/negative) - Motivation during conduction |  |
| **Biography exercises**   - Expressed/perceived difficulties - Difficulty level appropriate - Comprehension issues - How was the exercise received? - Reactions (positive/negative) - Motivation during conduction |  |

1. **Communication and Relationship Between Participant and Caregiver**

| **Communication** How did the communication between participant and caregiver go? |  |
| --- | --- |
| **Mood & mood changes** What was the mood between participant and caregiver?  Were there moments when the mood changed? |  |
| **Support** To what extent was support from the caregiver needed and accepted? |  |
| **Attitude of the caregiver toward the user** How was the caregiver's attitude toward the participant? |  |
| **Joint participation** Which contents were carried out together? |  |
| **Conversation stimulus** Which contents stimulated conversation between the participant and the caregiver? |  |
| **General impression / reaction** After completing the exercise? |  |
